# Duodenal Obstruction as First Presentation of Metastatic Breast Cancer

**DOI:** 10.1155/2015/605719

**Published:** 2015-07-21

**Authors:** Sami Khairy, Ayman Azzam, Shamayel Mohammed, Kausar Suleman, Abdurahman Khawaji, Tarek Amin

**Affiliations:** ^1^Division of Neurosurgery, Department of Surgery, King Abdulaziz Medical City, P.O. Box 22490, Riyadh 11426, Saudi Arabia; ^2^Department of General Surgery, Faculty of Medicine, Alexandria University, Alexandria, Egypt; ^3^Department of Pathology and Laboratory Medicine, King Faisal Specialist Hospital and Research Center (KFSH&RC), P.O. Box 3354, Riyadh 11211, Saudi Arabia; ^4^Department Medical Oncology, King Faisal Specialist Hospital and Research Center (KFSH&RC), P.O. Box 3354, Riyadh 11211, Saudi Arabia; ^5^Department of Radiology, King Faisal Specialist Hospital and Research Center (KFSH&RC), P.O. Box 3354, Riyadh 11211, Saudi Arabia; ^6^Department of Surgical Oncology, King Faisal Specialist Hospital and Research Center (KFSH&RC), P.O. Box 3354, Riyadh 11211, Saudi Arabia

## Abstract

The metastatic breast cancer to the duodenum is rare in spite of common breast cancer. In this paper, we are reporting a rare case of 50-year-old lady who presented with intestinal obstruction as result of metastatic breast cancer which completely responds to chemotherapy. The tumor presents again as brain metastasis after stop of Herceptin due to cardiac toxicity.

## 1. Introduction

Breast cancer is the most common cancer among females worldwide and in Saudi Arabia. According to official cancer registry in Saudi Arabia it accounts for 22.4% of all female cancers [1–3]. Usually the presentation of primary breast lesion starts as local or regional symptoms that can be noticed by the patient herself or her healthcare provider. In case of metastatic breast cancer, the presentation behaves according to site of metastasis. Lungs, bones, liver, and brain are considered the most common sites of breast metastasis while gastrointestinal tract is still very rare to involve [4].

Here, we are reporting a case of 50-year-old woman who presented with intestinal obstruction from duodenal mass as initial presentation of metastatic breast cancer.

## 2. Case Presentation

A 50-year-old lady was referred to us as a case of duodenal cancer with gastric outlet obstruction. She presented with history of persistent vomiting for the last two months, which was bilious and increased in the severity over last few days. This vomiting was aggravated by oral intake and associated with abdominal pain. There is no distention nether change in the bowel habits. She does not have a significant past medical or surgical history.

On examination, she looked conscious, oriented, and vitally stable. Abdomen was soft, lax and not tender with normal bowel sounds. The initial blood work shows elevated white blood cells, low hemoglobin, and high platelets. Liver function test showed that alkaline phosphatase was 243 IU/L, Gamma-glutamyl transpeptidase was 296 IU/L, and amylase was 32 IU/L. Other lab analyses were within normal range.

**Figure 1:**
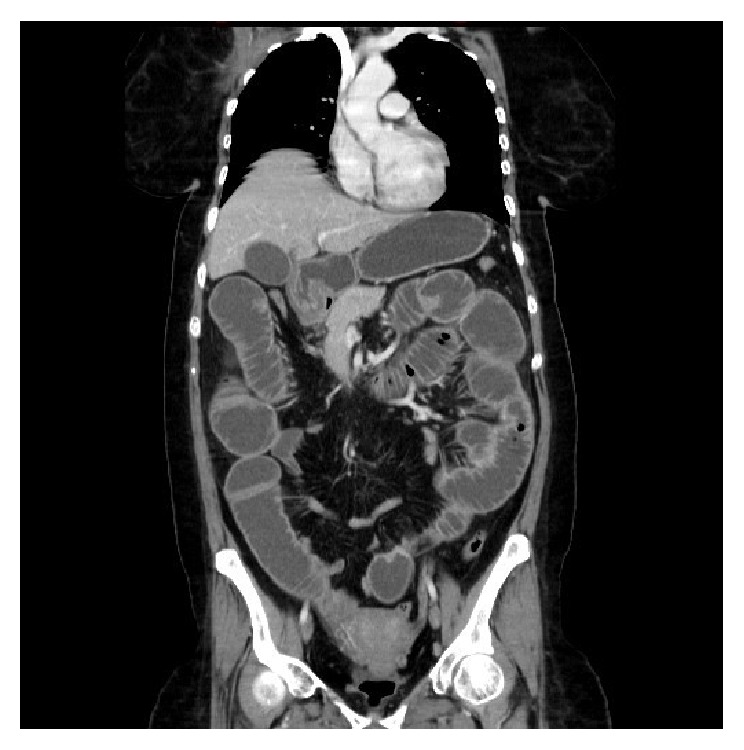
Enhanced coronal CT scan of the abdomen demonstrates mild circumferential wall thickening with luminal narrowing involving the second part of the duodenum associated with surrounding fat stranding.

The patient underwent upper gastrointestinal (GI) endoscopy which showed an ulcer in the gastric area and the duodenal area. Multiple biopsies taken from duodenum showed poorly differentiated adenocarcinoma. Computed tomography (CT) scan was carried out and showed mild wall thickening of the duodenum with narrow lumen about middle long segment of the 2nd part. Also a right axillary ill-defined mass was found with infiltration of the adjacent fat and no enlarged lymph nodes in the pelvis or the abdomen (Figure 1). For that, a mammogram was done, which showed predominant fatty involvement, which was uncertain.

**Figure 2:**
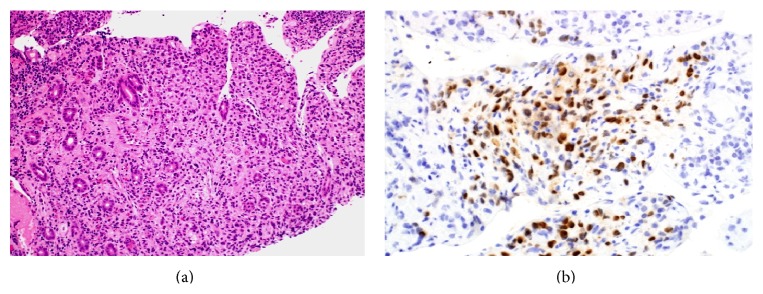
(a) Section from the duodenal biopsy showed infiltration of the lamina propria by poorly differentiated neoplastic cells with rare cells exhibiting intracytoplasmic lumen with mucin or eccentric nuclei suggesting a poorly differentiated adenocarcinoma. The neoplastic cells where positive for CK (AE1/AE3), CK7, and (b) ER and negative for PR. The morphology as well as immunohistochemical studies were consistent with poorly differentiated adenocarcinoma.

**Figure 3:**
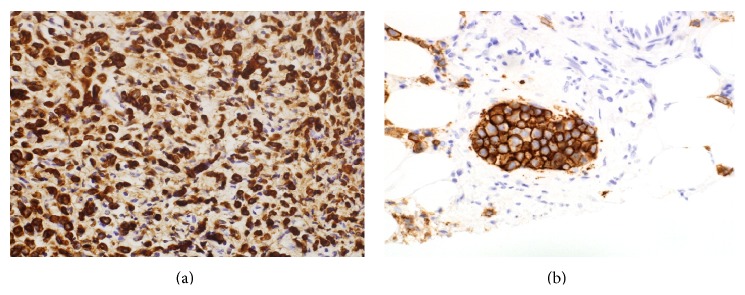
(a) Biopsy from the axillary mass shows infiltration by similar poorly differentiated neoplastic cells. The cells where positive for (b) HER2/neu. The overall findings were consistent with poorly differentiated adenocarcinoma of primary breast origin, most likely invasive ductal carcinoma.

She had a Positron emission tomography scan as well, which showed two foci of increased activity involving the C-loop of the duodenum suggestive of duodenal carcinoma with hyperactive nodal disease noted in the right axillary region. Ultrasound guided biopsy was taken from right axillary lymph node and showed poorly differentiated carcinoma with positive estrogen receptor (ER). Immunohistochemistry study also done for the previous biopsies from the duodenum showed strong positivity for ER and positivity for human epidermal growth factor receptor 2 (HER2) (Figures 2 and 3). The overall pathological findings were consistent with poorly differentiated adenocarcinoma of primary breast origin, most likely invasive ductal carcinoma.

**Figure 4:**
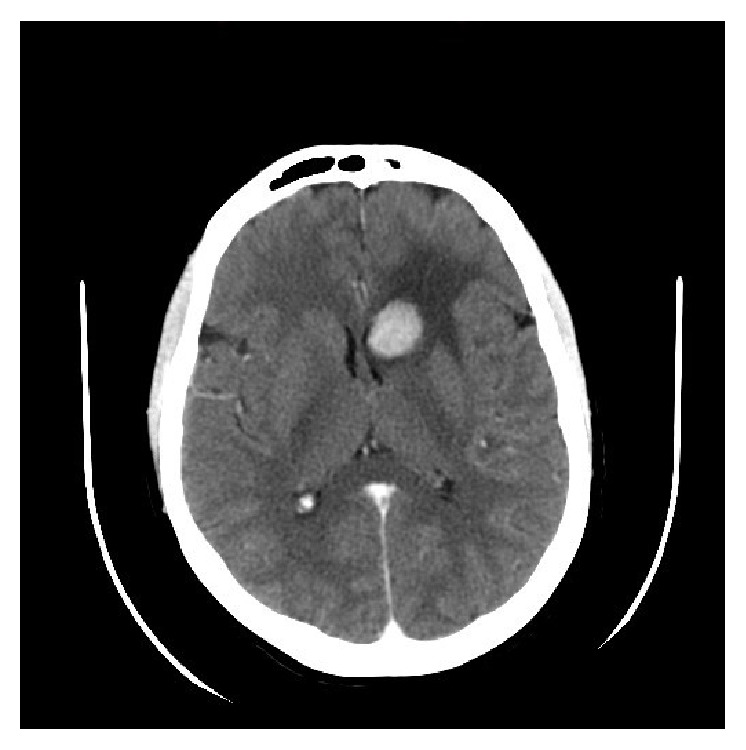
Enhanced axial CT scan of the brain shows multiple enhancing brain lesions seen supratentorially and infratentorially; the largest is seen in left caudate head with surrounding reactive parenchymal edema.

The patient underwent tumor resection and gastrojejunostomy anastomosis. After recovery from surgery, she was started on systemic chemotherapy Taxotere with Herceptin for 6 cycles. Afterwards, CT showed no residual of tumor and showed disappearing of auxiliary mass. So the patient was switched to Femara and Herceptin. During the treatment with Herceptin, she required stopping it few times and manage her cardiac ejection fraction EF decreased. Finally after 4 months of start of Herceptin, we required stopping it due to 20% drop in EF (35–40%) and symptomatic heart failure though patient was on antifailure treatment. She received a total of 25 doses of Herceptin. Three months later, she presented to emergency department with severe headache and CT showed brain metastases for which she received radiation therapy to brain (Figure 4).

Seven months after radiation therapy, she developed intestinal obstruction and she was admitted with diffuse peritoneal metastases and she had laparotomy as palliative surgery. Now she is on palliative chemotherapy with weekly Taxol and Herceptin as her EF is improved (50%).

## 3. Discussion

The presentation of GI metastasis from breast cancer is usually not specific to origin and the common signs to present with are abdominal pain, nausea, vomiting, alternation of bowl habit, and bleeding. All these signs and symptoms mimic the primary intestinal disorder and it is difficult to distinguish it from breast cancer metastasis clinically [5–7].

The intestinal obstruction is rare to be an initial presentation of invasive breast cancer without other signs [8, 9]. Borst and Ingold study shows that less than one percentage of metastatic breast cancers had gastrointestinal metastasis and most of these cases were invasive lobular carcinoma [10]. The metastasis of invasive ductal carcinoma commonly involves the liver, lung, and brain, while the invasive lobular carcinoma mainly metastasizes to gynecological organs and gastrointestinal tract [11].

In Annals of Surgical Oncology, McLemore et al. published the presentation of breast cancer in gastrointestinal tract, which showed that the colon and rectum are the most common sites of GI track for metastasis with 45% followed by 28% in stomach, 19% in small intestine, and 8% in the esophagus [12]. The GI metastasis from breast is indication of poor prognosis [5].

Surgical management was found to be helpful in survival of limited cases with limited GI metastasis, but overall the survival benefit is not proven [10, 11]. Even with surgical intervention, no patient survived after 5 years in Mourra et al. study with small survival advantage in patients who had surgery comparing with no surgery patients [5].

In our case, duodenal obstruction was the first manifestation of metastatic breast cancer without current breast signs or symptoms and without family history of breast cancer. This presentation is rare but should be considered.

In this case, the metastasis initially was to duodenum, which is not common in invasive ductal carcinoma as in invasive lobular carcinoma. The clinical presentation of GI metastasis alone is not enough to diagnose the origin. So the imaging and histopathology diagnostic tools are found to be essential in diagnosis. The early diagnosis and treatment are strongly associated with improvement of the outcome.

## Appendix

See Figures 1, 2, 3, and 4.
